# Crystal structure of {6,6′-dibenzoyl-4,4′-di-*tert*-butyl-2,2′-[(ethane-1,2-di­yl)di­nitrilo­bis­(phenyl­methanylyl­idene)]diphenolato-κ^4^ *O* ^1^,*N*,*N*′,*O* ^1′^}nickel(II)

**DOI:** 10.1107/S2056989015021052

**Published:** 2015-11-11

**Authors:** Abhishek K. Gupta, Ray J. Butcher, Anjan Sil

**Affiliations:** a Department of Metallurgical and Materials Engineering, Indian Institute of Technology Roorkee, Roorkee 247 667, India; b Department of Chemistry, Howard University, 525 College Street NW, Washington, DC 20059, USA

**Keywords:** crystal structure, synthesis, Ni^II^ complex, N_2_O_2_ Schiff base, C—H⋯O hydrogen bonding

## Abstract

The mononuclear title complex, [Ni(C_50_H_46_N_2_O_4_)], crystallizes in the triclinic space group *P*-1, with two mol­ecules in the asymmetric unit (*Z*′ = 2). Each Ni^II^ atom has a slightly distorted square-planar geometry [ω = 3.91 (5)° and 2.04 (7)°] defined by the two phenolate O and two imine N atoms of the tetra­dentate Schiff base ligand. The dihedral angles between the central phenolate ring and peripheral phenyl rings are 60.5 (2)/70.0 (2) and 86.4 (2)/56.1 (2)° in mol­ecule *A*, and 89.43 (19)/18.0 (2) and 63.87 (19)/68.2 (2)° in mol­ecule *B*. The two central phenolate rings are twisted by angles of 19.37 (19) and 19.36 (18)° in the two mol­ecules. The packing is stabilized through intra- and inter­molecular C—H⋯O and C—H⋯π inter­actions, forming chains parallel to the *b* axis. The *tert*-butyl groups in one of the two mol­ecules are positionally disordered with a refined occupancy ratio of 0.707 (13):0.293 (13).

## Abstract 2

The title compound crystallized with two independent mol­ecules in the asymmetric unit. In the crystal, mol­ecules are linked by weak intra- and inter­molecular C—H⋯O and C—H⋯π inter­actions, forming chains along the *b* axis.

## Chemical context   

Transition metal Schiff base complexes with N_2_O_2_ donor sets have attracted much attention in material science (Sukwattanasinitt *et al.*, 2003 ▸; Thurston *et al.*, 2003 ▸), catalysis (Martín *et al.*, 2015 ▸; Gupta & Sutar, 2008 ▸; Cozzi, 2004 ▸) and in drug design (Sun *et al.*, 2007 ▸). Very recently, a Ni^II^ complex with a tetra­dentate Schiff base having an oxidized methyl­ene arm has been described (Gupta *et al.*, 2015 ▸). We herein report the synthesis and crystal structure of an Ni^II^ complex with a similar tetra­dentate Schiff base containing an unoxidized methyl­ene arm.
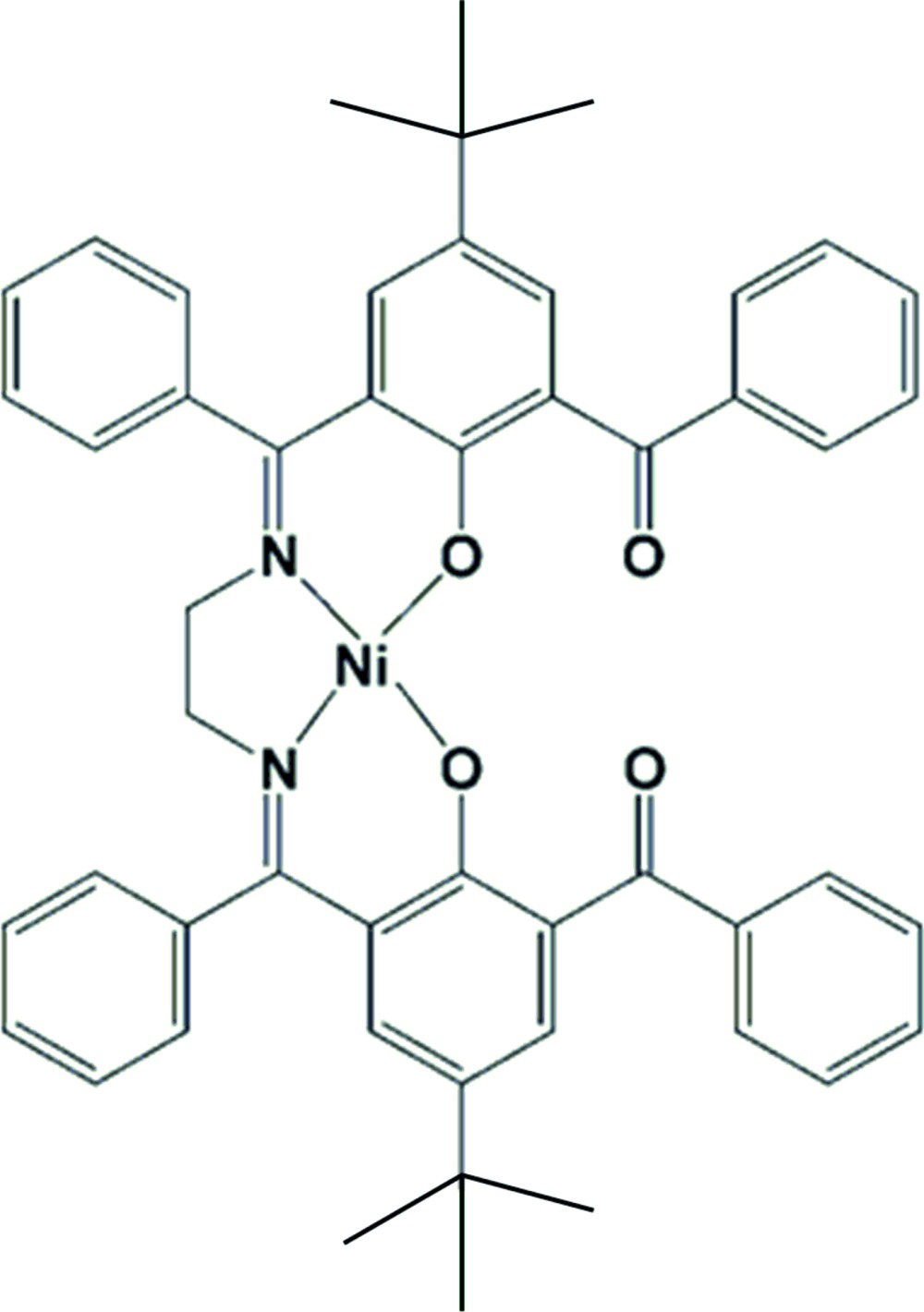


## Structural commentary   

**Figure 1:**
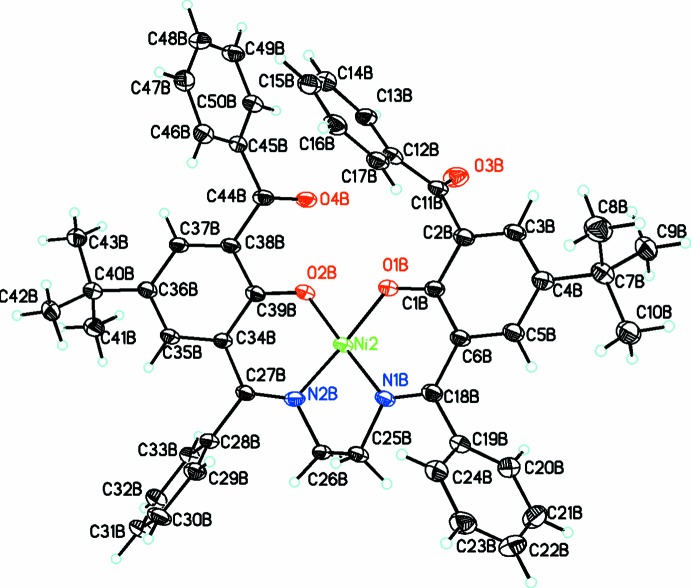
The mol­ecular structure of one of the independent mol­ecules (*B*) of the title compound, showing the atom labelling. Displacement ellipsoids are drawn at the 30% probability level.

The mononuclear title complex crystallizes in the triclinic space group *P* 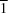, with two mol­ecules in the asymmetric unit (*Z*′ = 2). The mol­ecular structure of one of the independent mol­ecules is shown in Fig. 1 ▸. The Ni^II^ atom is coordinated by a tetra­dentate dianionic ligand involving two phenolato O and two imine N atoms. The coordination geometry around the metal atom is slightly distorted square planar [β = 88.44 (16) and 88.55 (15)° (half of the angle N1*A*—Ni1—O2*A*/N2*A*—Ni1—O1*A* in *A* or N1*B*—Ni2—O2*B*/ N2*B*—Ni2—O1*B* in B) and ω = 3.8 (4) and 2.2 (4)° (the angle between the coordination planes Ni1–N1*A*–O1*A* and Ni1–N2*A*–O2*A* in *A* and Ni2–N1*B*–O1*B* and Ni2–N2*B*–O2*B* in *B*) for Ni1 and Ni2, respectively (Rybak-Akimova *et al.*, 2001 ▸)]. The Ni^II^ atom deviates from the coordination plane by 0.046 (4) and 0.006 (3) Å, respectively. Atoms C25 and C26 in *A* and *B* are significantly out of plane, as indicated by the N—C—C—N torsion angles, −32.9 (7)° in *A* and −40.5 (3)° in *B*. The dihedral angles between the central phenolato ring (C1*A*–C6*A* & C34*A*–C39*A*; C1*B*–C6*B* & C34*B*–C39*B*) and the peripheral phenyl rings (C12*A*–C17*A*; C19*A*–C24*A*; C28*A*–C33*A*; C45*A*–C50*A* and C12*B*–C17*B*; C19*B*–C24*B*; C28*B*–C33*B*; C45*B*–C50*B*) are 60.5 (2) & 70.0 (2)° and 86.4 (2) & 56.1 (2)° in mol­ecule *A* and 89.43 (19) & 18.0 (2)° and 63.87 (19) & 68.2 (2)° in mol­ecule *B*. The two central phenolato rings (C1*A*–C6*A* & C34*A*–C39*A* and C1*B*–C6*B* & C34*B*–C39*B*) are twisted by angles of 19.37 (19) and 19.36 (18)°, respectively, in the two mol­ecules.

## Supra­molecular features   

**Figure 2:**
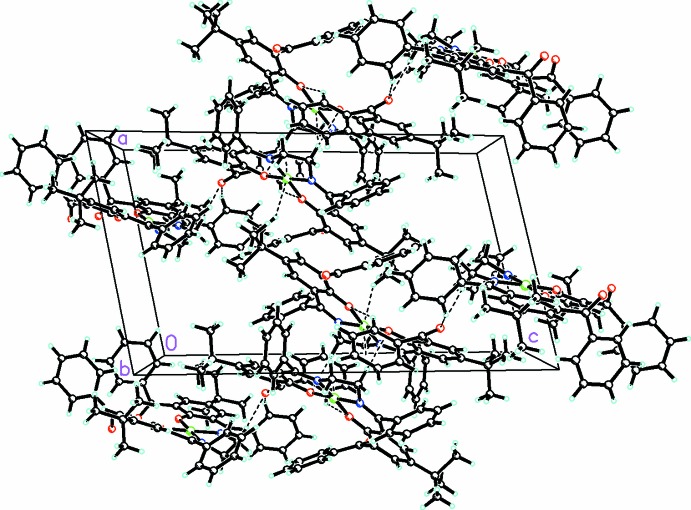
Packing diagram of [Ni(C_50_H_46_N_2_O_2_)], viewed along *b* axis. Dashed lines indicate weak C—H⋯O inter­molecular inter­actions (see Table 1 ▸ for details).

In the crystal, mol­ecules are linked by intra­molecular C—H⋯O hydrogen bonds. These are further linked into chains extending parallel to the *b* axis by weak inter­molecular C—H⋯O and C—H⋯π inter­actions (Table 1 ▸ and Fig. 2 ▸).

## Database survey   

A search of the Cambridge Structural Database (CSD, Version 5.35, last update November 2014; Groom & Allen, 2014 ▸) for the basic skeleton of this compound gave no hits.

## Synthesis and crystallization   

An ethano­lic solution of NiCl_2_·6H_2_O (0.474 g, 2.00 mmol) was added dropwise to the stirred hot solution of the tetra­dentate Schiff base, *L*H_2_ (Gupta *et al.*, 2015 ▸), bis­[2-{6-benzoyl-4-*tert*-butyl­phenol}benzimido­yl]-1,2-ethane 1.48 g, 2.00 mmol) in ethanol under argon. The resulting wine-red solution was heated to reflux at 343–353 K. The clear solution thus obtained was filtered and allowed to cool to ambient temperature. Slow evaporation of the solvent resulted in red–brown irregular plate-shaped crystals within a few days (yield: 0.80 g, 50%; m.p. 570–573 K). Analysis calculated for C_50_H_46_N_2_O_4_Ni (%): C 75.29, H 5.81. Found: C 75.40, H 5.60.

## Refinement   

Crystal data, data collection and structure refinement details are summarized in Table 2 ▸. All H atoms were positioned geometrically and allowed to ride on their parent atoms, with C—H = 0.95–0.99 Å with *U*_iso_(H) = 1.5*U*_eq_(C) for methyl H atoms and = 1.2*U*_eq_(C) for other H atoms. The refined occupancy ratios for the disordered *t*-butyl groups are 0.707 (13):0.293 (13) for atoms C8*A*/C9*A*/C10*A* and C8*C*/C9*C*/C10*C* in mol­ecule *A*. The ISOR restraint and EADP constraint commands in the *SHELXL2014* software were used for the disordered atoms. There are voids in the structure of 348 Å^3^ due to the packing of the mol­ecules but when these were checked using the SQUEEZE routine (Spek, 2015 ▸) an electron count per cell of only one electron resulted, and SQUEEZE was not used to correct for residual electron density within these voids.

## Supplementary Material

Crystal structure: contains datablock(s) I. DOI: 10.1107/S2056989015021052/zl2639sup1.cif

Structure factors: contains datablock(s) I. DOI: 10.1107/S2056989015021052/zl2639Isup2.hkl

CCDC reference: 1435344

Additional supporting information:  crystallographic information; 3D view; checkCIF report

## Figures and Tables

**Table 1:** Hydrogen-bond geometry (Å, °) *Cg*2, *Cg*4, *Cg*8, *Cg*11, *Cg*13, *Cg*14 and *Cg*17 are the centroids of the Ni1/O1*A*/C1*A*/C6*A*/C18*A*/N1*A*, C1*A*–C6*A*, C34*A*–C39*A*, Ni2/O1*B*/C1*B*/C6*B*/C18*B*/N1*B*, C1*B*–C6*B*, C12*B*–C17*B* and C34*B*–C39*B* rings, respectively.

*D*—H⋯*A*	*D*—H	H⋯*A*	*D*⋯*A*	*D*—H⋯*A*
C50*A*—H50*A*⋯O1*A*	0.95	2.55	3.354 (5)	143
C25*A*—H25*B*⋯O2*A* ^i^	0.99	2.40	3.394 (7)	178
C24*B*—H24*B*⋯O4*A*	0.95	2.58	3.343 (6)	138
C25*B*—H25*C*⋯O4*A*	0.99	2.35	3.336 (5)	171
C26*B*—H26*C*⋯O1*B* ^ii^	0.99	2.45	3.294 (5)	143
C26*B*—H26*C*⋯O2*B* ^ii^	0.99	2.45	3.309 (5)	144
C25*B*—H25*D*⋯O4*B* ^ii^	0.99	2.45	3.283 (4)	141
C10*A*—H10*C*⋯*Cg*2^iii^	0.98	2.98	3.771 (12)	138
C15*B*—H15*B*⋯*Cg*11^iv^	0.95	2.74	3.593 (5)	150
C20*A*—H20*A*⋯*Cg*8^i^	0.95	2.77	3.424 (5)	127
C20*B*—H20*B*⋯*Cg*17^ii^	0.95	2.71	3.585 (5)	153
C33*A*—H33*A*⋯*Cg*4^i^	0.95	2.79	3.623 (5)	146
C33*B*—H33*B*⋯*Cg*13^ii^	0.95	2.66	3.465 (4)	143
C43*A*—H43*B*⋯*Cg*14^iv^	0.98	2.60	3.457 (5)	147

Symmetry codes: (i) 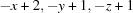; (ii) 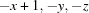; (iii) 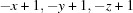; (iv) 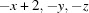.

**Table 2:** Experimental details

Crystal data	
Chemical formula	[Ni(C_50_H_46_N_2_O_4_)]
*M*_r_	797.60
Crystal system, space group	Triclinic, *P* 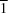
Temperature (K)	120
*a*, *b*, *c* (Å)	12.4401 (9), 16.8987 (11), 21.5185 (12)
α, β, γ (°)	100.843 (5), 100.029 (6), 99.890 (6)
*V* (Å^3^)	4276.1 (5)
*Z*	4
Radiation type	Cu *K*α
μ (mm^−1^)	1.02
Crystal size (mm)	0.29 × 0.10 × 0.02

Data collection	
Diffractometer	Agilent SuperNova Dual Source diffractometer with an Atlas detector
Absorption correction	Multi-scan
*T*_min_, *T*_max_	0.736, 1.000
No. of measured, independent and observed [*I* > 2σ(*I*)] reflections	31365, 17306, 9944
*R*_int_	0.088
(sin θ/λ)_max_ (Å^−1^)	0.630

Refinement	
*R*[*F* ^2^ > 2σ(*F* ^2^)], *wR*(*F* ^2^), *S*	0.068, 0.208, 1.02
No. of reflections	17306
No. of parameters	1076
No. of restraints	42
H-atom treatment	H-atom parameters not refined
Δρ_max_, Δρ_min_ (e Å^−3^)	0.57, −0.58

Computer programs: *CrysAlis PRO* (Agilent, 2013 ▸), *SIR92* (Altomare *et al.*, 1993 ▸), *SHELXL2014* (Sheldrick, 2015 ▸), *SHELXTL* (Sheldrick, 2008 ▸) and *publCIF* (Westrip, 2010 ▸).

## supplementary crystallographic information

### Crystal data

**Table:**

[Ni(C_50_H_46_N_2_O_4_)]	*Z* = 4
*M**_r_* = 797.60	*F*(000) = 1680
Triclinic, *P*1	*D*_x_ = 1.239 Mg m^−^^3^
*a* = 12.4401 (9) Å	Cu *K*α radiation, λ = 1.54184 Å
*b* = 16.8987 (11) Å	Cell parameters from 3725 reflections
*c* = 21.5185 (12) Å	θ = 2.7–76.1°
α = 100.843 (5)°	µ = 1.02 mm^−^^1^
β = 100.029 (6)°	*T* = 120 K
γ = 99.890 (6)°	Plate, red-brown
*V* = 4276.1 (5) Å^3^	0.29 × 0.10 × 0.02 mm

### Data collection

**Table:**

Agilent SuperNova Dual Source diffractometer with an Atlas detector	9944 reflections with *I* > 2σ(*I*)
Detector resolution: 10.6501 pixels mm^-1^	*R*_int_ = 0.088
ω scans	θ_max_ = 76.3°, θ_min_ = 2.7°
Absorption correction: multi-scan	*h* = −9→15
*T*_min_ = 0.736, *T*_max_ = 1.000	*k* = −21→20
31365 measured reflections	*l* = −26→25
17306 independent reflections	

### Refinement

**Table:**

Refinement on *F*^2^	42 restraints
Least-squares matrix: full	Hydrogen site location: inferred from neighbouring sites
*R*[*F*^2^ > 2σ(*F*^2^)] = 0.068	H-atom parameters not refined
*wR*(*F*^2^) = 0.208	*w* = 1/[σ^2^(*F*_o_^2^) + (0.0554*P*)^2^] where *P* = (*F*_o_^2^ + 2*F*_c_^2^)/3
*S* = 1.02	(Δ/σ)_max_ < 0.001
17306 reflections	Δρ_max_ = 0.57 e Å^−^^3^
1076 parameters	Δρ_min_ = −0.58 e Å^−^^3^

### Special details

**Table:**

Geometry. All e.s.d.'s (except the e.s.d. in the dihedral angle between two l.s. planes) are estimated using the full covariance matrix. The cell e.s.d.'s are taken into account individually in the estimation of e.s.d.'s in distances, angles and torsion angles; correlations between e.s.d.'s in cell parameters are only used when they are defined by crystal symmetry. An approximate (isotropic) treatment of cell e.s.d.'s is used for estimating e.s.d.'s involving l.s. planes.

### Fractional atomic coordinates and isotropic or equivalent isotropic displacement parameters (Å^2^)

**Table:**

	*x*	*y*	*z*	*U*_iso_*/*U*_eq_	Occ. (<1)
Ni1	0.83716 (6)	0.46720 (4)	0.44208 (3)	0.03896 (17)	
O1A	0.7458 (2)	0.39067 (19)	0.46952 (16)	0.0466 (7)	
O2A	0.8545 (2)	0.37825 (17)	0.38296 (14)	0.0424 (7)	
O3A	0.6021 (3)	0.1934 (2)	0.50874 (19)	0.0632 (10)	
O4A	0.8316 (3)	0.1593 (2)	0.25874 (17)	0.0564 (9)	
N1A	0.8237 (3)	0.5544 (2)	0.50501 (18)	0.0432 (8)	
N2A	0.9237 (3)	0.5434 (2)	0.41045 (17)	0.0417 (8)	
C1A	0.6790 (3)	0.4034 (3)	0.5095 (2)	0.0421 (9)	
C2A	0.6050 (3)	0.3340 (3)	0.5175 (2)	0.0423 (9)	
C3A	0.5419 (4)	0.3420 (3)	0.5652 (2)	0.0445 (10)	
H3AA	0.4978	0.2937	0.5718	0.053*	
C4A	0.5415 (4)	0.4180 (3)	0.6033 (2)	0.0456 (10)	
C5A	0.6092 (4)	0.4867 (3)	0.5928 (2)	0.0455 (10)	
H5AA	0.6086	0.5394	0.6175	0.055*	
C6A	0.6784 (4)	0.4823 (3)	0.5477 (2)	0.0419 (9)	
C7A	0.4817 (9)	0.4258 (7)	0.6603 (6)	0.0529 (14)	0.707 (18)
C8A	0.4139 (13)	0.3441 (6)	0.6640 (7)	0.094 (4)	0.707 (18)
H8AA	0.4644	0.3097	0.6789	0.141*	0.707 (18)
H8AB	0.3685	0.3161	0.6210	0.141*	0.707 (18)
H8AC	0.3649	0.3537	0.6945	0.141*	0.707 (18)
C9A	0.5672 (9)	0.4614 (10)	0.7232 (4)	0.081 (3)	0.707 (18)
H9AA	0.6083	0.5158	0.7221	0.121*	0.707 (18)
H9AB	0.6195	0.4247	0.7287	0.121*	0.707 (18)
H9AC	0.5291	0.4666	0.7595	0.121*	0.707 (18)
C10A	0.4000 (8)	0.4831 (6)	0.6509 (6)	0.052 (2)	0.707 (18)
H10A	0.4417	0.5382	0.6519	0.078*	0.707 (18)
H10B	0.3586	0.4864	0.6858	0.078*	0.707 (18)
H10C	0.3475	0.4612	0.6091	0.078*	0.707 (18)
C7C	0.468 (2)	0.4256 (15)	0.6536 (13)	0.0529 (14)	0.293 (18)
C8C	0.3582 (19)	0.3631 (18)	0.6310 (14)	0.086 (6)	0.293 (18)
H8CA	0.3274	0.3610	0.5854	0.129*	0.293 (18)
H8CB	0.3054	0.3793	0.6573	0.129*	0.293 (18)
H8CC	0.3707	0.3086	0.6356	0.129*	0.293 (18)
C9C	0.525 (2)	0.4092 (19)	0.7167 (11)	0.073 (6)	0.293 (18)
H9CA	0.5870	0.4555	0.7386	0.110*	0.293 (18)
H9CB	0.5531	0.3585	0.7078	0.110*	0.293 (18)
H9CC	0.4709	0.4029	0.7446	0.110*	0.293 (18)
C10C	0.434 (3)	0.5088 (16)	0.6654 (16)	0.073 (7)	0.293 (18)
H10D	0.4984	0.5529	0.6690	0.110*	0.293 (18)
H10E	0.4062	0.5170	0.7056	0.110*	0.293 (18)
H10F	0.3745	0.5098	0.6291	0.110*	0.293 (18)
C11A	0.5951 (4)	0.2480 (3)	0.4794 (2)	0.0469 (10)	
C12A	0.5620 (4)	0.2277 (3)	0.4075 (2)	0.0460 (10)	
C13A	0.5386 (4)	0.2861 (3)	0.3724 (2)	0.0512 (11)	
H13A	0.5495	0.3422	0.3940	0.061*	
C14A	0.4993 (4)	0.2626 (4)	0.3056 (2)	0.0571 (12)	
H14A	0.4850	0.3028	0.2816	0.068*	
C15A	0.4808 (4)	0.1798 (4)	0.2741 (2)	0.0590 (14)	
H15A	0.4504	0.1631	0.2288	0.071*	
C16A	0.5067 (4)	0.1224 (3)	0.3087 (2)	0.0553 (12)	
H16A	0.4967	0.0664	0.2869	0.066*	
C17A	0.5469 (4)	0.1456 (3)	0.3748 (3)	0.0531 (12)	
H17A	0.5645	0.1054	0.3982	0.064*	
C18A	0.7549 (3)	0.5555 (3)	0.5443 (2)	0.0422 (9)	
C19A	0.7552 (3)	0.6361 (3)	0.5886 (2)	0.0420 (9)	
C20A	0.8042 (4)	0.6529 (3)	0.6537 (2)	0.0469 (10)	
H20A	0.8357	0.6126	0.6716	0.056*	
C21A	0.8072 (4)	0.7292 (3)	0.6931 (2)	0.0509 (11)	
H21A	0.8400	0.7402	0.7379	0.061*	
C22A	0.7639 (4)	0.7884 (3)	0.6683 (2)	0.0492 (11)	
H22A	0.7674	0.8403	0.6956	0.059*	
C23A	0.7148 (4)	0.7720 (3)	0.6029 (3)	0.0518 (11)	
H23A	0.6843	0.8129	0.5853	0.062*	
C24A	0.7102 (4)	0.6956 (3)	0.5627 (2)	0.0466 (10)	
H24A	0.6764	0.6844	0.5179	0.056*	
C25A	0.9115 (4)	0.6259 (3)	0.5109 (3)	0.0675 (17)	
H25A	0.8868	0.6768	0.5281	0.081*	
H25B	0.9792	0.6230	0.5415	0.081*	
C26A	0.9373 (6)	0.6285 (3)	0.4482 (3)	0.0701 (17)	
H26A	1.0150	0.6590	0.4538	0.084*	
H26B	0.8866	0.6575	0.4248	0.084*	
C27A	0.9632 (3)	0.5327 (3)	0.3577 (2)	0.0399 (9)	
C28A	1.0236 (4)	0.6066 (3)	0.3388 (2)	0.0416 (9)	
C29A	0.9624 (4)	0.6537 (3)	0.3071 (3)	0.0524 (11)	
H29A	0.8833	0.6370	0.2946	0.063*	
C30A	1.0164 (5)	0.7258 (3)	0.2934 (3)	0.0600 (13)	
H30A	0.9743	0.7574	0.2707	0.072*	
C31A	1.1318 (5)	0.7516 (3)	0.3128 (3)	0.0593 (13)	
H31A	1.1688	0.8015	0.3049	0.071*	
C32A	1.1917 (4)	0.7037 (3)	0.3438 (3)	0.0546 (12)	
H32A	1.2707	0.7203	0.3563	0.066*	
C33A	1.1381 (4)	0.6312 (3)	0.3570 (2)	0.0505 (11)	
H33A	1.1805	0.5987	0.3785	0.061*	
C34A	0.9509 (3)	0.4523 (3)	0.3165 (2)	0.0388 (9)	
C35A	0.9859 (3)	0.4474 (2)	0.25703 (19)	0.0359 (8)	
H35A	1.0167	0.4969	0.2459	0.043*	
C36A	0.9767 (3)	0.3726 (3)	0.21426 (19)	0.0365 (8)	
C37A	0.9354 (3)	0.3015 (2)	0.23339 (19)	0.0356 (8)	
H37A	0.9305	0.2498	0.2053	0.043*	
C38A	0.9006 (3)	0.3020 (2)	0.2917 (2)	0.0367 (8)	
C39A	0.9014 (3)	0.3791 (3)	0.3333 (2)	0.0385 (9)	
C40A	1.0098 (3)	0.3714 (3)	0.1484 (2)	0.0396 (9)	
C41A	0.9664 (4)	0.4383 (3)	0.1188 (2)	0.0458 (10)	
H41A	0.8854	0.4299	0.1149	0.069*	
H41B	0.9837	0.4354	0.0758	0.069*	
H41C	1.0024	0.4926	0.1467	0.069*	
C42A	1.1390 (4)	0.3888 (3)	0.1596 (2)	0.0481 (10)	
H42A	1.1616	0.3939	0.1188	0.072*	
H42B	1.1664	0.3432	0.1747	0.072*	
H42C	1.1707	0.4402	0.1922	0.072*	
C43A	0.9608 (4)	0.2885 (3)	0.1010 (2)	0.0491 (11)	
H43A	0.8791	0.2778	0.0937	0.074*	
H43B	0.9881	0.2448	0.1191	0.074*	
H43C	0.9837	0.2898	0.0598	0.074*	
C44A	0.8682 (3)	0.2185 (3)	0.3046 (2)	0.0411 (9)	
C45A	0.8956 (4)	0.1989 (3)	0.3706 (2)	0.0420 (9)	
C46A	0.9714 (5)	0.1478 (3)	0.3775 (3)	0.0591 (13)	
H46A	1.0073	0.1317	0.3431	0.071*	
C47A	0.9950 (5)	0.1202 (4)	0.4337 (3)	0.0690 (15)	
H47A	1.0482	0.0864	0.4382	0.083*	
C48A	0.9411 (5)	0.1418 (4)	0.4834 (3)	0.0642 (14)	
H48A	0.9570	0.1225	0.5220	0.077*	
C49A	0.8639 (4)	0.1916 (3)	0.4770 (2)	0.0532 (12)	
H49A	0.8263	0.2058	0.5110	0.064*	
C50A	0.8415 (4)	0.2206 (3)	0.4208 (2)	0.0474 (10)	
H50A	0.7893	0.2553	0.4167	0.057*	
Ni2	0.64422 (5)	0.02378 (4)	0.03150 (3)	0.03342 (16)	
O1B	0.6904 (2)	−0.06962 (16)	−0.00047 (13)	0.0356 (6)	
O2B	0.6654 (2)	0.04983 (17)	−0.04492 (14)	0.0374 (6)	
O3B	0.6786 (3)	−0.2282 (2)	−0.12420 (16)	0.0516 (8)	
O4B	0.6574 (2)	−0.00410 (18)	−0.17764 (15)	0.0442 (7)	
N1B	0.6272 (3)	−0.00554 (19)	0.10936 (16)	0.0337 (7)	
N2B	0.6027 (3)	0.1218 (2)	0.06147 (16)	0.0351 (7)	
C1B	0.7008 (3)	−0.1311 (2)	0.02600 (19)	0.0333 (8)	
C2B	0.7367 (3)	−0.1976 (2)	−0.0102 (2)	0.0364 (8)	
C3B	0.7525 (3)	−0.2653 (2)	0.0139 (2)	0.0377 (9)	
H3BA	0.774 (4)	−0.310 (3)	−0.011 (2)	0.045*	
C4B	0.7326 (3)	−0.2740 (3)	0.0759 (2)	0.0403 (9)	
C5B	0.6954 (3)	−0.2098 (2)	0.1100 (2)	0.0359 (8)	
H5BA	0.6802	−0.2141	0.1511	0.043*	
C6B	0.6784 (3)	−0.1384 (2)	0.08773 (19)	0.0334 (8)	
C7B	0.7527 (4)	−0.3498 (3)	0.1001 (2)	0.0444 (10)	
C8B	0.8765 (4)	−0.3543 (3)	0.1057 (3)	0.0593 (13)	
H8BA	0.9233	−0.3058	0.1372	0.089*	
H8BB	0.8960	−0.3558	0.0634	0.089*	
H8BC	0.8889	−0.4043	0.1202	0.089*	
C9B	0.6797 (4)	−0.4277 (3)	0.0526 (3)	0.0533 (12)	
H9BA	0.6008	−0.4258	0.0497	0.080*	
H9BB	0.6944	−0.4766	0.0681	0.080*	
H9BC	0.6975	−0.4305	0.0097	0.080*	
C10B	0.7235 (4)	−0.3487 (3)	0.1664 (2)	0.0509 (11)	
H10G	0.7678	−0.2989	0.1976	0.076*	
H10H	0.7401	−0.3976	0.1812	0.076*	
H10I	0.6439	−0.3489	0.1630	0.076*	
C11B	0.7523 (3)	−0.1913 (2)	−0.0778 (2)	0.0366 (8)	
C12B	0.8582 (3)	−0.1429 (2)	−0.0849 (2)	0.0375 (9)	
C13B	0.8664 (3)	−0.1287 (3)	−0.1462 (2)	0.0400 (9)	
H13B	0.8027	−0.1464	−0.1809	0.048*	
C14B	0.9673 (4)	−0.0889 (3)	−0.1567 (2)	0.0490 (11)	
H14B	0.9730	−0.0804	−0.1985	0.059*	
C15B	1.0597 (4)	−0.0618 (3)	−0.1052 (3)	0.0497 (11)	
H15B	1.1288	−0.0347	−0.1120	0.060*	
C16B	1.0513 (4)	−0.0741 (3)	−0.0436 (2)	0.0466 (10)	
H16B	1.1142	−0.0552	−0.0085	0.056*	
C17B	0.9508 (3)	−0.1140 (3)	−0.0341 (2)	0.0415 (9)	
H17B	0.9451	−0.1219	0.0079	0.050*	
C18B	0.6429 (3)	−0.0722 (2)	0.12768 (19)	0.0343 (8)	
C19B	0.6281 (3)	−0.0819 (2)	0.1939 (2)	0.0362 (8)	
C20B	0.5364 (4)	−0.1373 (3)	0.2006 (2)	0.0439 (9)	
H20B	0.4843	−0.1702	0.1632	0.053*	
C21B	0.5210 (4)	−0.1447 (3)	0.2614 (3)	0.0545 (12)	
H21B	0.4584	−0.1825	0.2658	0.065*	
C22B	0.5973 (5)	−0.0966 (4)	0.3165 (3)	0.0628 (14)	
H22B	0.5862	−0.1012	0.3583	0.075*	
C23B	0.6888 (5)	−0.0423 (3)	0.3101 (2)	0.0563 (12)	
H23B	0.7412	−0.0099	0.3475	0.068*	
C24B	0.7041 (4)	−0.0350 (3)	0.2489 (2)	0.0447 (10)	
H24B	0.7673	0.0024	0.2446	0.054*	
C25B	0.5951 (3)	0.0607 (2)	0.1536 (2)	0.0371 (8)	
H25C	0.6622	0.0953	0.1849	0.045*	
H25D	0.5428	0.0364	0.1781	0.045*	
C26B	0.5404 (3)	0.1118 (2)	0.11314 (19)	0.0351 (8)	
H26C	0.4615	0.0842	0.0938	0.042*	
H26D	0.5418	0.1664	0.1403	0.042*	
C27B	0.6237 (3)	0.1901 (2)	0.04261 (18)	0.0319 (8)	
C28B	0.6056 (3)	0.2685 (2)	0.08168 (19)	0.0354 (8)	
C29B	0.6700 (3)	0.2986 (3)	0.1440 (2)	0.0407 (9)	
H29B	0.7221	0.2689	0.1609	0.049*	
C30B	0.6590 (4)	0.3719 (3)	0.1818 (2)	0.0498 (11)	
H30B	0.7032	0.3924	0.2244	0.060*	
C31B	0.5829 (4)	0.4148 (3)	0.1568 (3)	0.0543 (12)	
H31B	0.5760	0.4655	0.1819	0.065*	
C32B	0.5168 (4)	0.3838 (3)	0.0952 (2)	0.0477 (10)	
H32B	0.4630	0.4127	0.0789	0.057*	
C33B	0.5282 (3)	0.3113 (2)	0.0572 (2)	0.0386 (9)	
H33B	0.4834	0.2908	0.0147	0.046*	
C34B	0.6691 (3)	0.1957 (2)	−0.01500 (19)	0.0345 (8)	
C35B	0.6944 (3)	0.2714 (2)	−0.03242 (19)	0.0355 (8)	
H35B	0.6865	0.3192	−0.0039	0.043*	
C36B	0.7305 (3)	0.2812 (2)	−0.0891 (2)	0.0357 (8)	
C37B	0.7471 (3)	0.2095 (2)	−0.12759 (19)	0.0347 (8)	
H37B	0.7759	0.2140	−0.1651	0.042*	
C38B	0.7238 (3)	0.1331 (2)	−0.11392 (19)	0.0342 (8)	
C39B	0.6836 (3)	0.1229 (2)	−0.05652 (18)	0.0326 (8)	
C40B	0.7464 (3)	0.3653 (3)	−0.1065 (2)	0.0394 (9)	
C41B	0.6334 (4)	0.3925 (3)	−0.1139 (2)	0.0476 (10)	
H41D	0.5777	0.3515	−0.1478	0.071*	
H41E	0.6420	0.4461	−0.1258	0.071*	
H41F	0.6087	0.3971	−0.0728	0.071*	
C42B	0.8322 (4)	0.4295 (3)	−0.0524 (2)	0.0444 (10)	
H42D	0.9059	0.4157	−0.0502	0.067*	
H42E	0.8100	0.4292	−0.0109	0.067*	
H42F	0.8352	0.4843	−0.0617	0.067*	
C43B	0.7848 (4)	0.3627 (3)	−0.1705 (2)	0.0458 (10)	
H43D	0.8567	0.3458	−0.1669	0.069*	
H43E	0.7929	0.4175	−0.1802	0.069*	
H43F	0.7293	0.3230	−0.2053	0.069*	
C44B	0.7284 (3)	0.0590 (3)	−0.16311 (19)	0.0360 (8)	
C45B	0.8236 (3)	0.0663 (2)	−0.1987 (2)	0.0365 (8)	
C46B	0.9307 (3)	0.1096 (3)	−0.1682 (2)	0.0429 (9)	
H46B	0.9455	0.1401	−0.1246	0.052*	
C47B	1.0168 (4)	0.1078 (3)	−0.2021 (3)	0.0505 (11)	
H47B	1.0906	0.1366	−0.1812	0.061*	
C48B	0.9955 (4)	0.0648 (3)	−0.2654 (3)	0.0528 (12)	
H48B	1.0543	0.0638	−0.2882	0.063*	
C49B	0.8890 (4)	0.0233 (3)	−0.2955 (2)	0.0510 (11)	
H49B	0.8740	−0.0052	−0.3396	0.061*	
C50B	0.8035 (4)	0.0223 (3)	−0.2626 (2)	0.0426 (9)	
H50B	0.7306	−0.0084	−0.2835	0.051*	

### Atomic displacement parameters (Å^2^)

**Table:**

	*U*^11^	*U*^22^	*U*^33^	*U*^12^	*U*^13^	*U*^23^
Ni1	0.0460 (4)	0.0365 (4)	0.0316 (4)	0.0105 (3)	0.0121 (3)	−0.0045 (3)
O1A	0.0506 (16)	0.0429 (17)	0.0426 (17)	0.0084 (13)	0.0200 (14)	−0.0075 (13)
O2A	0.0573 (16)	0.0348 (15)	0.0354 (15)	0.0080 (12)	0.0215 (13)	−0.0007 (12)
O3A	0.094 (3)	0.0438 (19)	0.050 (2)	0.0103 (18)	0.0256 (19)	0.0005 (16)
O4A	0.075 (2)	0.0388 (17)	0.0435 (19)	−0.0038 (15)	0.0143 (16)	−0.0057 (14)
N1A	0.0395 (17)	0.044 (2)	0.0379 (19)	0.0076 (15)	0.0075 (15)	−0.0085 (15)
N2A	0.058 (2)	0.0315 (17)	0.0313 (17)	0.0113 (15)	0.0106 (15)	−0.0071 (14)
C1A	0.044 (2)	0.047 (2)	0.032 (2)	0.0133 (18)	0.0117 (17)	−0.0054 (17)
C2A	0.047 (2)	0.043 (2)	0.036 (2)	0.0107 (18)	0.0155 (18)	−0.0033 (17)
C3A	0.050 (2)	0.047 (2)	0.036 (2)	0.0124 (19)	0.0150 (18)	0.0030 (18)
C4A	0.055 (2)	0.049 (2)	0.033 (2)	0.014 (2)	0.0186 (19)	−0.0011 (18)
C5A	0.058 (2)	0.047 (2)	0.033 (2)	0.019 (2)	0.0162 (19)	−0.0035 (18)
C6A	0.050 (2)	0.045 (2)	0.029 (2)	0.0159 (18)	0.0088 (17)	−0.0024 (17)
C7A	0.068 (4)	0.066 (3)	0.037 (3)	0.030 (3)	0.027 (3)	0.011 (2)
C8A	0.154 (10)	0.070 (5)	0.089 (8)	0.037 (5)	0.091 (8)	0.022 (4)
C9A	0.077 (5)	0.151 (10)	0.033 (3)	0.058 (6)	0.026 (3)	0.018 (4)
C10A	0.044 (4)	0.073 (5)	0.044 (5)	0.019 (4)	0.019 (3)	0.009 (4)
C7C	0.068 (4)	0.066 (3)	0.037 (3)	0.030 (3)	0.027 (3)	0.011 (2)
C8C	0.075 (8)	0.107 (11)	0.072 (13)	0.016 (8)	0.042 (7)	−0.013 (9)
C9C	0.077 (11)	0.091 (15)	0.059 (8)	0.018 (10)	0.022 (7)	0.031 (9)
C10C	0.100 (18)	0.084 (9)	0.056 (15)	0.042 (10)	0.049 (12)	0.015 (8)
C11A	0.054 (2)	0.045 (2)	0.040 (2)	0.0075 (19)	0.0168 (19)	−0.0015 (19)
C12A	0.046 (2)	0.045 (2)	0.040 (2)	0.0016 (18)	0.0175 (18)	−0.0082 (19)
C13A	0.058 (3)	0.053 (3)	0.036 (2)	0.004 (2)	0.022 (2)	−0.007 (2)
C14A	0.060 (3)	0.068 (3)	0.039 (3)	0.006 (2)	0.016 (2)	0.002 (2)
C15A	0.050 (2)	0.075 (4)	0.037 (2)	0.000 (2)	0.016 (2)	−0.020 (2)
C16A	0.061 (3)	0.054 (3)	0.043 (3)	0.012 (2)	0.018 (2)	−0.013 (2)
C17A	0.055 (3)	0.049 (3)	0.050 (3)	0.011 (2)	0.018 (2)	−0.008 (2)
C18A	0.043 (2)	0.043 (2)	0.035 (2)	0.0109 (17)	0.0082 (17)	−0.0074 (17)
C19A	0.0390 (19)	0.042 (2)	0.038 (2)	0.0099 (17)	0.0091 (17)	−0.0088 (18)
C20A	0.052 (2)	0.046 (2)	0.038 (2)	0.0182 (19)	0.0050 (19)	−0.0055 (19)
C21A	0.054 (2)	0.051 (3)	0.039 (2)	0.014 (2)	0.0106 (19)	−0.015 (2)
C22A	0.046 (2)	0.044 (2)	0.052 (3)	0.0084 (19)	0.020 (2)	−0.008 (2)
C23A	0.055 (2)	0.047 (3)	0.056 (3)	0.018 (2)	0.023 (2)	0.002 (2)
C24A	0.049 (2)	0.047 (2)	0.043 (2)	0.0117 (19)	0.0143 (19)	0.0030 (19)
C25A	0.063 (3)	0.059 (3)	0.064 (4)	−0.002 (2)	0.030 (3)	−0.028 (3)
C26A	0.116 (5)	0.034 (2)	0.056 (3)	0.004 (3)	0.041 (3)	−0.012 (2)
C27A	0.045 (2)	0.034 (2)	0.037 (2)	0.0087 (16)	0.0091 (17)	−0.0017 (16)
C28A	0.054 (2)	0.035 (2)	0.033 (2)	0.0095 (17)	0.0145 (18)	−0.0045 (16)
C29A	0.055 (3)	0.046 (3)	0.055 (3)	0.010 (2)	0.013 (2)	0.007 (2)
C30A	0.077 (3)	0.048 (3)	0.057 (3)	0.016 (2)	0.013 (3)	0.015 (2)
C31A	0.088 (4)	0.040 (2)	0.049 (3)	0.007 (2)	0.028 (3)	0.001 (2)
C32A	0.060 (3)	0.050 (3)	0.049 (3)	0.000 (2)	0.015 (2)	0.005 (2)
C33A	0.060 (3)	0.047 (3)	0.044 (3)	0.014 (2)	0.013 (2)	0.005 (2)
C34A	0.044 (2)	0.034 (2)	0.035 (2)	0.0078 (16)	0.0111 (17)	−0.0006 (16)
C35A	0.045 (2)	0.0342 (19)	0.0277 (19)	0.0067 (16)	0.0113 (16)	0.0035 (15)
C36A	0.0402 (19)	0.039 (2)	0.0282 (19)	0.0109 (16)	0.0079 (15)	−0.0020 (15)
C37A	0.0366 (18)	0.036 (2)	0.0304 (19)	0.0088 (15)	0.0082 (15)	−0.0040 (15)
C38A	0.0377 (18)	0.035 (2)	0.034 (2)	0.0052 (15)	0.0107 (16)	−0.0012 (16)
C39A	0.0394 (19)	0.040 (2)	0.032 (2)	0.0076 (16)	0.0110 (16)	−0.0022 (16)
C40A	0.047 (2)	0.037 (2)	0.034 (2)	0.0121 (17)	0.0136 (17)	0.0002 (16)
C41A	0.052 (2)	0.052 (3)	0.033 (2)	0.014 (2)	0.0121 (18)	0.0046 (18)
C42A	0.050 (2)	0.048 (3)	0.049 (3)	0.0124 (19)	0.019 (2)	0.008 (2)
C43A	0.066 (3)	0.041 (2)	0.039 (2)	0.007 (2)	0.026 (2)	−0.0019 (19)
C44A	0.042 (2)	0.034 (2)	0.045 (2)	0.0057 (16)	0.0181 (18)	−0.0023 (17)
C45A	0.049 (2)	0.034 (2)	0.039 (2)	0.0069 (17)	0.0113 (18)	−0.0010 (17)
C46A	0.072 (3)	0.055 (3)	0.061 (3)	0.028 (2)	0.034 (3)	0.010 (2)
C47A	0.084 (4)	0.074 (4)	0.065 (4)	0.043 (3)	0.024 (3)	0.026 (3)
C48A	0.085 (4)	0.062 (3)	0.053 (3)	0.025 (3)	0.017 (3)	0.021 (3)
C49A	0.067 (3)	0.051 (3)	0.040 (2)	0.008 (2)	0.022 (2)	0.000 (2)
C50A	0.046 (2)	0.042 (2)	0.051 (3)	0.0063 (18)	0.018 (2)	−0.002 (2)
Ni2	0.0397 (3)	0.0303 (3)	0.0299 (3)	0.0080 (3)	0.0148 (3)	−0.0014 (3)
O1B	0.0424 (14)	0.0338 (14)	0.0321 (14)	0.0079 (11)	0.0162 (11)	0.0033 (11)
O2B	0.0467 (14)	0.0326 (14)	0.0343 (14)	0.0107 (11)	0.0181 (12)	−0.0002 (11)
O3B	0.0465 (16)	0.062 (2)	0.0368 (17)	−0.0005 (14)	0.0119 (13)	−0.0035 (15)
O4B	0.0461 (15)	0.0409 (16)	0.0397 (16)	0.0010 (13)	0.0193 (13)	−0.0073 (13)
N1B	0.0364 (15)	0.0312 (16)	0.0333 (17)	0.0083 (12)	0.0144 (13)	−0.0008 (13)
N2B	0.0369 (15)	0.0399 (18)	0.0277 (16)	0.0059 (13)	0.0152 (13)	0.0004 (13)
C1B	0.0325 (17)	0.0325 (19)	0.0313 (19)	0.0026 (14)	0.0115 (14)	−0.0024 (15)
C2B	0.0339 (17)	0.037 (2)	0.037 (2)	0.0076 (15)	0.0117 (15)	−0.0003 (16)
C3B	0.0395 (19)	0.034 (2)	0.038 (2)	0.0119 (16)	0.0135 (16)	−0.0025 (16)
C4B	0.044 (2)	0.039 (2)	0.039 (2)	0.0086 (17)	0.0157 (17)	0.0028 (17)
C5B	0.0372 (18)	0.0323 (19)	0.038 (2)	0.0088 (15)	0.0125 (16)	0.0023 (16)
C6B	0.0348 (17)	0.0325 (19)	0.0295 (19)	0.0059 (14)	0.0093 (15)	−0.0022 (15)
C7B	0.050 (2)	0.036 (2)	0.046 (2)	0.0118 (18)	0.0145 (19)	0.0019 (18)
C8B	0.059 (3)	0.062 (3)	0.065 (3)	0.031 (2)	0.014 (2)	0.017 (3)
C9B	0.075 (3)	0.034 (2)	0.054 (3)	0.013 (2)	0.022 (2)	0.006 (2)
C10B	0.067 (3)	0.046 (2)	0.045 (3)	0.020 (2)	0.016 (2)	0.010 (2)
C11B	0.0396 (19)	0.0297 (18)	0.041 (2)	0.0092 (15)	0.0178 (17)	−0.0017 (16)
C12B	0.047 (2)	0.0300 (19)	0.035 (2)	0.0091 (16)	0.0163 (17)	−0.0038 (15)
C13B	0.045 (2)	0.037 (2)	0.037 (2)	0.0082 (17)	0.0158 (17)	0.0001 (16)
C14B	0.062 (3)	0.042 (2)	0.048 (3)	0.010 (2)	0.027 (2)	0.008 (2)
C15B	0.043 (2)	0.044 (2)	0.059 (3)	0.0067 (18)	0.021 (2)	−0.002 (2)
C16B	0.044 (2)	0.039 (2)	0.048 (3)	0.0024 (17)	0.0115 (19)	−0.0086 (19)
C17B	0.044 (2)	0.037 (2)	0.041 (2)	0.0101 (17)	0.0156 (18)	−0.0053 (17)
C18B	0.0323 (17)	0.0324 (19)	0.033 (2)	0.0016 (14)	0.0103 (15)	−0.0023 (15)
C19B	0.0426 (19)	0.0339 (19)	0.033 (2)	0.0113 (16)	0.0130 (16)	0.0011 (15)
C20B	0.046 (2)	0.044 (2)	0.044 (2)	0.0105 (18)	0.0174 (19)	0.0088 (19)
C21B	0.060 (3)	0.060 (3)	0.053 (3)	0.014 (2)	0.027 (2)	0.021 (2)
C22B	0.084 (4)	0.070 (4)	0.042 (3)	0.017 (3)	0.029 (3)	0.015 (2)
C23B	0.084 (3)	0.054 (3)	0.030 (2)	0.019 (3)	0.010 (2)	0.004 (2)
C24B	0.053 (2)	0.040 (2)	0.040 (2)	0.0091 (18)	0.0135 (19)	0.0025 (18)
C25B	0.043 (2)	0.0329 (19)	0.036 (2)	0.0071 (16)	0.0191 (17)	−0.0011 (16)
C26B	0.0425 (19)	0.0329 (19)	0.0301 (19)	0.0064 (15)	0.0196 (16)	−0.0018 (15)
C27B	0.0313 (16)	0.0327 (19)	0.0289 (18)	0.0059 (14)	0.0106 (14)	−0.0033 (14)
C28B	0.0408 (19)	0.0312 (19)	0.034 (2)	0.0062 (15)	0.0177 (16)	0.0002 (15)
C29B	0.041 (2)	0.044 (2)	0.032 (2)	0.0075 (17)	0.0106 (16)	−0.0034 (17)
C30B	0.057 (3)	0.045 (2)	0.039 (2)	0.009 (2)	0.016 (2)	−0.0119 (19)
C31B	0.067 (3)	0.040 (2)	0.056 (3)	0.015 (2)	0.028 (2)	−0.008 (2)
C32B	0.054 (2)	0.038 (2)	0.056 (3)	0.0164 (19)	0.023 (2)	0.005 (2)
C33B	0.042 (2)	0.037 (2)	0.037 (2)	0.0085 (16)	0.0155 (17)	0.0027 (16)
C34B	0.0370 (18)	0.0347 (19)	0.0299 (19)	0.0042 (15)	0.0148 (15)	−0.0015 (15)
C35B	0.0423 (19)	0.0319 (19)	0.0317 (19)	0.0093 (15)	0.0149 (16)	−0.0023 (15)
C36B	0.0376 (18)	0.034 (2)	0.034 (2)	0.0079 (15)	0.0124 (16)	0.0010 (16)
C37B	0.0377 (18)	0.036 (2)	0.0294 (19)	0.0055 (15)	0.0144 (15)	0.0008 (15)
C38B	0.0374 (18)	0.0351 (19)	0.0278 (18)	0.0084 (15)	0.0123 (15)	−0.0040 (15)
C39B	0.0354 (17)	0.0333 (19)	0.0282 (18)	0.0083 (14)	0.0099 (14)	0.0010 (15)
C40B	0.046 (2)	0.036 (2)	0.037 (2)	0.0079 (17)	0.0171 (17)	0.0031 (16)
C41B	0.059 (3)	0.042 (2)	0.048 (3)	0.017 (2)	0.019 (2)	0.0108 (19)
C42B	0.055 (2)	0.032 (2)	0.041 (2)	0.0009 (17)	0.0145 (19)	−0.0001 (17)
C43B	0.058 (2)	0.041 (2)	0.040 (2)	0.0091 (19)	0.0183 (19)	0.0069 (18)
C44B	0.0394 (19)	0.040 (2)	0.0267 (18)	0.0084 (16)	0.0098 (15)	−0.0002 (16)
C45B	0.046 (2)	0.0300 (19)	0.035 (2)	0.0081 (16)	0.0179 (17)	0.0042 (15)
C46B	0.042 (2)	0.039 (2)	0.046 (2)	0.0070 (17)	0.0151 (18)	0.0011 (18)
C47B	0.041 (2)	0.040 (2)	0.074 (3)	0.0092 (18)	0.022 (2)	0.011 (2)
C48B	0.058 (3)	0.049 (3)	0.068 (3)	0.023 (2)	0.040 (3)	0.016 (2)
C49B	0.068 (3)	0.047 (3)	0.045 (3)	0.019 (2)	0.030 (2)	0.005 (2)
C50B	0.050 (2)	0.037 (2)	0.041 (2)	0.0102 (17)	0.0176 (18)	0.0006 (17)

### Geometric parameters (Å, º)

**Table:**

Ni1—O1A	1.824 (3)	C47A—H47A	0.9500
Ni1—N2A	1.842 (4)	C48A—C49A	1.387 (7)
Ni1—O2A	1.847 (3)	C48A—H48A	0.9500
Ni1—N1A	1.856 (3)	C49A—C50A	1.391 (8)
O1A—C1A	1.312 (5)	C49A—H49A	0.9500
O2A—C39A	1.305 (5)	C50A—H50A	0.9500
O3A—C11A	1.218 (7)	Ni2—O1B	1.826 (3)
O4A—C44A	1.223 (5)	Ni2—O2B	1.830 (3)
N1A—C18A	1.304 (6)	Ni2—N2B	1.853 (3)
N1A—C25A	1.453 (6)	Ni2—N1B	1.870 (3)
N2A—C27A	1.308 (6)	O1B—C1B	1.290 (5)
N2A—C26A	1.478 (5)	O2B—C39B	1.295 (5)
C1A—C2A	1.419 (6)	O3B—C11B	1.215 (5)
C1A—C6A	1.429 (6)	O4B—C44B	1.212 (5)
C2A—C3A	1.396 (6)	N1B—C18B	1.296 (6)
C2A—C11A	1.501 (6)	N1B—C25B	1.483 (4)
C3A—C4A	1.390 (6)	N2B—C27B	1.295 (5)
C3A—H3AA	0.9500	N2B—C26B	1.481 (4)
C4A—C5A	1.390 (7)	C1B—C2B	1.426 (5)
C4A—C7C	1.53 (3)	C1B—C6B	1.427 (5)
C4A—C7A	1.539 (14)	C2B—C3B	1.371 (6)
C5A—C6A	1.406 (6)	C2B—C11B	1.521 (6)
C5A—H5AA	0.9500	C3B—C4B	1.429 (6)
C6A—C18A	1.446 (6)	C3B—H3BA	0.95 (5)
C7A—C8A	1.512 (12)	C4B—C5B	1.386 (5)
C7A—C9A	1.516 (11)	C4B—C7B	1.513 (6)
C7A—C10A	1.532 (10)	C5B—C6B	1.415 (6)
C8A—H8AA	0.9800	C5B—H5BA	0.9500
C8A—H8AB	0.9800	C6B—C18B	1.458 (5)
C8A—H8AC	0.9800	C7B—C10B	1.531 (6)
C9A—H9AA	0.9800	C7B—C9B	1.537 (6)
C9A—H9AB	0.9800	C7B—C8B	1.540 (6)
C9A—H9AC	0.9800	C8B—H8BA	0.9800
C10A—H10A	0.9800	C8B—H8BB	0.9800
C10A—H10B	0.9800	C8B—H8BC	0.9800
C10A—H10C	0.9800	C9B—H9BA	0.9800
C7C—C8C	1.516 (18)	C9B—H9BB	0.9800
C7C—C9C	1.516 (18)	C9B—H9BC	0.9800
C7C—C10C	1.527 (17)	C10B—H10G	0.9800
C8C—H8CA	0.9800	C10B—H10H	0.9800
C8C—H8CB	0.9800	C10B—H10I	0.9800
C8C—H8CC	0.9800	C11B—C12B	1.475 (5)
C9C—H9CA	0.9800	C12B—C17B	1.389 (6)
C9C—H9CB	0.9800	C12B—C13B	1.403 (6)
C9C—H9CC	0.9800	C13B—C14B	1.394 (6)
C10C—H10D	0.9800	C13B—H13B	0.9500
C10C—H10E	0.9800	C14B—C15B	1.394 (7)
C10C—H10F	0.9800	C14B—H14B	0.9500
C11A—C12A	1.487 (7)	C15B—C16B	1.399 (7)
C12A—C13A	1.391 (8)	C15B—H15B	0.9500
C12A—C17A	1.398 (6)	C16B—C17B	1.382 (6)
C13A—C14A	1.391 (7)	C16B—H16B	0.9500
C13A—H13A	0.9500	C17B—H17B	0.9500
C14A—C15A	1.397 (7)	C18B—C19B	1.503 (6)
C14A—H14A	0.9500	C19B—C24B	1.387 (6)
C15A—C16A	1.376 (9)	C19B—C20B	1.393 (6)
C15A—H15A	0.9500	C20B—C21B	1.381 (7)
C16A—C17A	1.379 (7)	C20B—H20B	0.9500
C16A—H16A	0.9500	C21B—C22B	1.394 (8)
C17A—H17A	0.9500	C21B—H21B	0.9500
C18A—C19A	1.508 (5)	C22B—C23B	1.378 (8)
C19A—C20A	1.381 (6)	C22B—H22B	0.9500
C19A—C24A	1.390 (7)	C23B—C24B	1.388 (7)
C20A—C21A	1.395 (6)	C23B—H23B	0.9500
C20A—H20A	0.9500	C24B—H24B	0.9500
C21A—C22A	1.362 (8)	C25B—C26B	1.499 (6)
C21A—H21A	0.9500	C25B—H25C	0.9900
C22A—C23A	1.389 (7)	C25B—H25D	0.9900
C22A—H22A	0.9500	C26B—H26C	0.9900
C23A—C24A	1.399 (6)	C26B—H26D	0.9900
C23A—H23A	0.9500	C27B—C34B	1.461 (5)
C24A—H24A	0.9500	C27B—C28B	1.503 (5)
C25A—C26A	1.446 (8)	C28B—C29B	1.388 (6)
C25A—H25A	0.9900	C28B—C33B	1.391 (6)
C25A—H25B	0.9900	C29B—C30B	1.391 (6)
C26A—H26A	0.9900	C29B—H29B	0.9500
C26A—H26B	0.9900	C30B—C31B	1.384 (8)
C27A—C34A	1.443 (5)	C30B—H30B	0.9500
C27A—C28A	1.504 (6)	C31B—C32B	1.385 (7)
C28A—C33A	1.377 (7)	C31B—H31B	0.9500
C28A—C29A	1.383 (7)	C32B—C33B	1.384 (6)
C29A—C30A	1.396 (7)	C32B—H32B	0.9500
C29A—H29A	0.9500	C33B—H33B	0.9500
C30A—C31A	1.390 (8)	C34B—C35B	1.398 (6)
C30A—H30A	0.9500	C34B—C39B	1.437 (5)
C31A—C32A	1.375 (8)	C35B—C36B	1.398 (6)
C31A—H31A	0.9500	C35B—H35B	0.9500
C32A—C33A	1.394 (7)	C36B—C37B	1.401 (5)
C32A—H32A	0.9500	C36B—C40B	1.528 (6)
C33A—H33A	0.9500	C37B—C38B	1.371 (6)
C34A—C35A	1.415 (6)	C37B—H37B	0.9500
C34A—C39A	1.422 (6)	C38B—C39B	1.439 (5)
C35A—C36A	1.391 (5)	C38B—C44B	1.497 (5)
C35A—H35A	0.9500	C40B—C43B	1.529 (6)
C36A—C37A	1.387 (6)	C40B—C42B	1.537 (6)
C36A—C40A	1.541 (6)	C40B—C41B	1.544 (6)
C37A—C38A	1.396 (6)	C41B—H41D	0.9800
C37A—H37A	0.9500	C41B—H41E	0.9800
C38A—C39A	1.433 (5)	C41B—H41F	0.9800
C38A—C44A	1.489 (6)	C42B—H42D	0.9800
C40A—C43A	1.525 (6)	C42B—H42E	0.9800
C40A—C41A	1.529 (6)	C42B—H42F	0.9800
C40A—C42A	1.550 (6)	C43B—H43D	0.9800
C41A—H41A	0.9800	C43B—H43E	0.9800
C41A—H41B	0.9800	C43B—H43F	0.9800
C41A—H41C	0.9800	C44B—C45B	1.519 (5)
C42A—H42A	0.9800	C45B—C46B	1.386 (6)
C42A—H42B	0.9800	C45B—C50B	1.392 (6)
C42A—H42C	0.9800	C46B—C47B	1.398 (6)
C43A—H43A	0.9800	C46B—H46B	0.9500
C43A—H43B	0.9800	C47B—C48B	1.376 (7)
C43A—H43C	0.9800	C47B—H47B	0.9500
C44A—C45A	1.514 (7)	C48B—C49B	1.370 (7)
C45A—C46A	1.391 (7)	C48B—H48B	0.9500
C45A—C50A	1.395 (6)	C49B—C50B	1.376 (6)
C46A—C47A	1.379 (8)	C49B—H49B	0.9500
C46A—H46A	0.9500	C50B—H50B	0.9500
C47A—C48A	1.385 (8)		
			
O1A—Ni1—N2A	176.99 (17)	C45A—C46A—H46A	119.6
O1A—Ni1—O2A	85.12 (13)	C46A—C47A—C48A	119.9 (5)
N2A—Ni1—O2A	93.85 (14)	C46A—C47A—H47A	120.0
O1A—Ni1—N1A	93.64 (15)	C48A—C47A—H47A	120.0
N2A—Ni1—N1A	87.52 (16)	C47A—C48A—C49A	120.1 (5)
O2A—Ni1—N1A	176.93 (17)	C47A—C48A—H48A	119.9
C1A—O1A—Ni1	128.0 (3)	C49A—C48A—H48A	119.9
C39A—O2A—Ni1	128.0 (3)	C48A—C49A—C50A	120.0 (4)
C18A—N1A—C25A	120.7 (4)	C48A—C49A—H49A	120.0
C18A—N1A—Ni1	128.9 (3)	C50A—C49A—H49A	120.0
C25A—N1A—Ni1	110.2 (3)	C49A—C50A—C45A	120.0 (5)
C27A—N2A—C26A	118.4 (4)	C49A—C50A—H50A	120.0
C27A—N2A—Ni1	129.0 (3)	C45A—C50A—H50A	120.0
C26A—N2A—Ni1	112.3 (3)	O1B—Ni2—O2B	83.46 (12)
O1A—C1A—C2A	118.0 (4)	O1B—Ni2—N2B	176.70 (13)
O1A—C1A—C6A	124.5 (4)	O2B—Ni2—N2B	93.76 (14)
C2A—C1A—C6A	117.5 (4)	O1B—Ni2—N1B	94.38 (13)
C3A—C2A—C1A	120.8 (4)	O2B—Ni2—N1B	177.56 (13)
C3A—C2A—C11A	116.1 (4)	N2B—Ni2—N1B	88.37 (14)
C1A—C2A—C11A	123.0 (4)	C1B—O1B—Ni2	128.1 (2)
C4A—C3A—C2A	122.2 (4)	C39B—O2B—Ni2	126.8 (2)
C4A—C3A—H3AA	118.9	C18B—N1B—C25B	120.1 (3)
C2A—C3A—H3AA	118.9	C18B—N1B—Ni2	128.3 (3)
C3A—C4A—C5A	116.8 (4)	C25B—N1B—Ni2	111.5 (3)
C3A—C4A—C7C	121.3 (10)	C27B—N2B—C26B	123.1 (3)
C5A—C4A—C7C	121.9 (10)	C27B—N2B—Ni2	127.6 (2)
C3A—C4A—C7A	122.0 (6)	C26B—N2B—Ni2	109.3 (3)
C5A—C4A—C7A	120.7 (5)	O1B—C1B—C2B	116.7 (3)
C4A—C5A—C6A	123.6 (4)	O1B—C1B—C6B	125.6 (3)
C4A—C5A—H5AA	118.2	C2B—C1B—C6B	117.7 (4)
C6A—C5A—H5AA	118.2	C3B—C2B—C1B	121.6 (4)
C5A—C6A—C1A	118.8 (4)	C3B—C2B—C11B	121.6 (3)
C5A—C6A—C18A	120.2 (4)	C1B—C2B—C11B	116.8 (4)
C1A—C6A—C18A	120.7 (4)	C2B—C3B—C4B	122.3 (4)
C8A—C7A—C9A	109.2 (10)	C2B—C3B—H3BA	121 (3)
C8A—C7A—C10A	107.0 (9)	C4B—C3B—H3BA	116 (3)
C9A—C7A—C10A	109.9 (8)	C5B—C4B—C3B	115.6 (4)
C8A—C7A—C4A	112.5 (8)	C5B—C4B—C7B	124.6 (4)
C9A—C7A—C4A	109.5 (8)	C3B—C4B—C7B	119.8 (4)
C10A—C7A—C4A	108.6 (8)	C4B—C5B—C6B	124.6 (4)
C7A—C8A—H8AA	109.5	C4B—C5B—H5BA	117.7
C7A—C8A—H8AB	109.5	C6B—C5B—H5BA	117.7
H8AA—C8A—H8AB	109.5	C5B—C6B—C1B	118.4 (3)
C7A—C8A—H8AC	109.5	C5B—C6B—C18B	121.1 (4)
H8AA—C8A—H8AC	109.5	C1B—C6B—C18B	120.5 (4)
H8AB—C8A—H8AC	109.5	C4B—C7B—C10B	111.8 (3)
C7A—C9A—H9AA	109.5	C4B—C7B—C9B	110.0 (4)
C7A—C9A—H9AB	109.5	C10B—C7B—C9B	107.7 (4)
H9AA—C9A—H9AB	109.5	C4B—C7B—C8B	109.6 (4)
C7A—C9A—H9AC	109.5	C10B—C7B—C8B	108.6 (4)
H9AA—C9A—H9AC	109.5	C9B—C7B—C8B	109.1 (4)
H9AB—C9A—H9AC	109.5	C7B—C8B—H8BA	109.5
C7A—C10A—H10A	109.5	C7B—C8B—H8BB	109.5
C7A—C10A—H10B	109.5	H8BA—C8B—H8BB	109.5
H10A—C10A—H10B	109.5	C7B—C8B—H8BC	109.5
C7A—C10A—H10C	109.5	H8BA—C8B—H8BC	109.5
H10A—C10A—H10C	109.5	H8BB—C8B—H8BC	109.5
H10B—C10A—H10C	109.5	C7B—C9B—H9BA	109.5
C8C—C7C—C9C	106.2 (19)	C7B—C9B—H9BB	109.5
C8C—C7C—C10C	104.2 (19)	H9BA—C9B—H9BB	109.5
C9C—C7C—C10C	111 (2)	C7B—C9B—H9BC	109.5
C8C—C7C—C4A	111.7 (18)	H9BA—C9B—H9BC	109.5
C9C—C7C—C4A	111.1 (18)	H9BB—C9B—H9BC	109.5
C10C—C7C—C4A	113 (2)	C7B—C10B—H10G	109.5
C7C—C8C—H8CA	109.5	C7B—C10B—H10H	109.5
C7C—C8C—H8CB	109.5	H10G—C10B—H10H	109.5
H8CA—C8C—H8CB	109.5	C7B—C10B—H10I	109.5
C7C—C8C—H8CC	109.5	H10G—C10B—H10I	109.5
H8CA—C8C—H8CC	109.5	H10H—C10B—H10I	109.5
H8CB—C8C—H8CC	109.5	O3B—C11B—C12B	122.2 (4)
C7C—C9C—H9CA	109.5	O3B—C11B—C2B	119.0 (4)
C7C—C9C—H9CB	109.5	C12B—C11B—C2B	118.8 (4)
H9CA—C9C—H9CB	109.5	C17B—C12B—C13B	119.1 (4)
C7C—C9C—H9CC	109.5	C17B—C12B—C11B	122.6 (4)
H9CA—C9C—H9CC	109.5	C13B—C12B—C11B	118.3 (4)
H9CB—C9C—H9CC	109.5	C14B—C13B—C12B	120.6 (4)
C7C—C10C—H10D	109.5	C14B—C13B—H13B	119.7
C7C—C10C—H10E	109.5	C12B—C13B—H13B	119.7
H10D—C10C—H10E	109.5	C15B—C14B—C13B	119.3 (5)
C7C—C10C—H10F	109.5	C15B—C14B—H14B	120.4
H10D—C10C—H10F	109.5	C13B—C14B—H14B	120.4
H10E—C10C—H10F	109.5	C14B—C15B—C16B	120.4 (4)
O3A—C11A—C12A	120.3 (4)	C14B—C15B—H15B	119.8
O3A—C11A—C2A	118.5 (4)	C16B—C15B—H15B	119.8
C12A—C11A—C2A	120.7 (4)	C17B—C16B—C15B	119.6 (4)
C13A—C12A—C17A	119.1 (5)	C17B—C16B—H16B	120.2
C13A—C12A—C11A	122.5 (4)	C15B—C16B—H16B	120.2
C17A—C12A—C11A	118.3 (5)	C16B—C17B—C12B	121.1 (4)
C14A—C13A—C12A	120.3 (5)	C16B—C17B—H17B	119.5
C14A—C13A—H13A	119.9	C12B—C17B—H17B	119.5
C12A—C13A—H13A	119.9	N1B—C18B—C6B	123.0 (4)
C13A—C14A—C15A	119.8 (6)	N1B—C18B—C19B	119.5 (3)
C13A—C14A—H14A	120.1	C6B—C18B—C19B	117.4 (4)
C15A—C14A—H14A	120.1	C24B—C19B—C20B	119.3 (4)
C16A—C15A—C14A	119.9 (5)	C24B—C19B—C18B	120.6 (4)
C16A—C15A—H15A	120.1	C20B—C19B—C18B	120.1 (4)
C14A—C15A—H15A	120.1	C21B—C20B—C19B	120.2 (5)
C15A—C16A—C17A	120.5 (5)	C21B—C20B—H20B	119.9
C15A—C16A—H16A	119.8	C19B—C20B—H20B	119.9
C17A—C16A—H16A	119.8	C20B—C21B—C22B	120.1 (5)
C16A—C17A—C12A	120.5 (6)	C20B—C21B—H21B	119.9
C16A—C17A—H17A	119.8	C22B—C21B—H21B	119.9
C12A—C17A—H17A	119.8	C23B—C22B—C21B	120.0 (5)
N1A—C18A—C6A	122.8 (4)	C23B—C22B—H22B	120.0
N1A—C18A—C19A	118.8 (4)	C21B—C22B—H22B	120.0
C6A—C18A—C19A	118.3 (4)	C22B—C23B—C24B	119.9 (5)
C20A—C19A—C24A	119.9 (4)	C22B—C23B—H23B	120.1
C20A—C19A—C18A	120.9 (4)	C24B—C23B—H23B	120.1
C24A—C19A—C18A	119.2 (4)	C19B—C24B—C23B	120.6 (5)
C19A—C20A—C21A	119.7 (5)	C19B—C24B—H24B	119.7
C19A—C20A—H20A	120.2	C23B—C24B—H24B	119.7
C21A—C20A—H20A	120.2	N1B—C25B—C26B	107.9 (3)
C22A—C21A—C20A	121.2 (5)	N1B—C25B—H25C	110.1
C22A—C21A—H21A	119.4	C26B—C25B—H25C	110.1
C20A—C21A—H21A	119.4	N1B—C25B—H25D	110.1
C21A—C22A—C23A	119.4 (4)	C26B—C25B—H25D	110.1
C21A—C22A—H22A	120.3	H25C—C25B—H25D	108.4
C23A—C22A—H22A	120.3	N2B—C26B—C25B	107.9 (3)
C22A—C23A—C24A	120.3 (5)	N2B—C26B—H26C	110.1
C22A—C23A—H23A	119.9	C25B—C26B—H26C	110.1
C24A—C23A—H23A	119.9	N2B—C26B—H26D	110.1
C19A—C24A—C23A	119.6 (5)	C25B—C26B—H26D	110.1
C19A—C24A—H24A	120.2	H26C—C26B—H26D	108.4
C23A—C24A—H24A	120.2	N2B—C27B—C34B	122.5 (3)
C26A—C25A—N1A	109.9 (4)	N2B—C27B—C28B	120.2 (3)
C26A—C25A—H25A	109.7	C34B—C27B—C28B	117.3 (3)
N1A—C25A—H25A	109.7	C29B—C28B—C33B	119.8 (4)
C26A—C25A—H25B	109.7	C29B—C28B—C27B	118.0 (4)
N1A—C25A—H25B	109.7	C33B—C28B—C27B	122.2 (4)
H25A—C25A—H25B	108.2	C28B—C29B—C30B	120.6 (4)
C25A—C26A—N2A	109.3 (5)	C28B—C29B—H29B	119.7
C25A—C26A—H26A	109.8	C30B—C29B—H29B	119.7
N2A—C26A—H26A	109.8	C31B—C30B—C29B	119.3 (5)
C25A—C26A—H26B	109.8	C31B—C30B—H30B	120.3
N2A—C26A—H26B	109.8	C29B—C30B—H30B	120.3
H26A—C26A—H26B	108.3	C30B—C31B—C32B	120.1 (4)
N2A—C27A—C34A	122.4 (4)	C30B—C31B—H31B	119.9
N2A—C27A—C28A	119.1 (4)	C32B—C31B—H31B	119.9
C34A—C27A—C28A	118.5 (4)	C33B—C32B—C31B	120.7 (5)
C33A—C28A—C29A	119.8 (5)	C33B—C32B—H32B	119.7
C33A—C28A—C27A	120.8 (4)	C31B—C32B—H32B	119.7
C29A—C28A—C27A	119.2 (4)	C32B—C33B—C28B	119.5 (4)
C28A—C29A—C30A	120.1 (5)	C32B—C33B—H33B	120.3
C28A—C29A—H29A	119.9	C28B—C33B—H33B	120.3
C30A—C29A—H29A	119.9	C35B—C34B—C39B	118.8 (3)
C31A—C30A—C29A	120.1 (5)	C35B—C34B—C27B	120.7 (3)
C31A—C30A—H30A	119.9	C39B—C34B—C27B	120.5 (4)
C29A—C30A—H30A	119.9	C34B—C35B—C36B	124.3 (3)
C32A—C31A—C30A	119.1 (5)	C34B—C35B—H35B	117.8
C32A—C31A—H31A	120.5	C36B—C35B—H35B	117.8
C30A—C31A—H31A	120.5	C35B—C36B—C37B	115.6 (4)
C31A—C32A—C33A	121.0 (5)	C35B—C36B—C40B	120.2 (3)
C31A—C32A—H32A	119.5	C37B—C36B—C40B	124.1 (4)
C33A—C32A—H32A	119.5	C38B—C37B—C36B	123.4 (4)
C28A—C33A—C32A	119.9 (5)	C38B—C37B—H37B	118.3
C28A—C33A—H33A	120.1	C36B—C37B—H37B	118.3
C32A—C33A—H33A	120.1	C37B—C38B—C39B	120.8 (3)
C35A—C34A—C39A	119.8 (4)	C37B—C38B—C44B	119.0 (3)
C35A—C34A—C27A	118.4 (4)	C39B—C38B—C44B	119.9 (4)
C39A—C34A—C27A	121.8 (4)	O2B—C39B—C34B	124.5 (3)
C36A—C35A—C34A	122.3 (4)	O2B—C39B—C38B	118.4 (3)
C36A—C35A—H35A	118.9	C34B—C39B—C38B	117.0 (4)
C34A—C35A—H35A	118.9	C36B—C40B—C43B	112.0 (3)
C37A—C36A—C35A	117.1 (4)	C36B—C40B—C42B	110.2 (4)
C37A—C36A—C40A	123.0 (3)	C43B—C40B—C42B	109.2 (3)
C35A—C36A—C40A	119.9 (4)	C36B—C40B—C41B	108.6 (3)
C36A—C37A—C38A	123.4 (4)	C43B—C40B—C41B	107.9 (4)
C36A—C37A—H37A	118.3	C42B—C40B—C41B	108.8 (3)
C38A—C37A—H37A	118.3	C40B—C41B—H41D	109.5
C37A—C38A—C39A	119.4 (4)	C40B—C41B—H41E	109.5
C37A—C38A—C44A	114.0 (3)	H41D—C41B—H41E	109.5
C39A—C38A—C44A	126.5 (4)	C40B—C41B—H41F	109.5
O2A—C39A—C34A	124.0 (4)	H41D—C41B—H41F	109.5
O2A—C39A—C38A	118.5 (4)	H41E—C41B—H41F	109.5
C34A—C39A—C38A	117.5 (4)	C40B—C42B—H42D	109.5
C43A—C40A—C41A	108.2 (4)	C40B—C42B—H42E	109.5
C43A—C40A—C36A	111.6 (4)	H42D—C42B—H42E	109.5
C41A—C40A—C36A	109.6 (3)	C40B—C42B—H42F	109.5
C43A—C40A—C42A	109.5 (3)	H42D—C42B—H42F	109.5
C41A—C40A—C42A	109.4 (4)	H42E—C42B—H42F	109.5
C36A—C40A—C42A	108.6 (4)	C40B—C43B—H43D	109.5
C40A—C41A—H41A	109.5	C40B—C43B—H43E	109.5
C40A—C41A—H41B	109.5	H43D—C43B—H43E	109.5
H41A—C41A—H41B	109.5	C40B—C43B—H43F	109.5
C40A—C41A—H41C	109.5	H43D—C43B—H43F	109.5
H41A—C41A—H41C	109.5	H43E—C43B—H43F	109.5
H41B—C41A—H41C	109.5	O4B—C44B—C38B	123.1 (3)
C40A—C42A—H42A	109.5	O4B—C44B—C45B	119.3 (3)
C40A—C42A—H42B	109.5	C38B—C44B—C45B	117.4 (3)
H42A—C42A—H42B	109.5	C46B—C45B—C50B	119.5 (4)
C40A—C42A—H42C	109.5	C46B—C45B—C44B	122.2 (4)
H42A—C42A—H42C	109.5	C50B—C45B—C44B	118.1 (4)
H42B—C42A—H42C	109.5	C45B—C46B—C47B	119.3 (4)
C40A—C43A—H43A	109.5	C45B—C46B—H46B	120.4
C40A—C43A—H43B	109.5	C47B—C46B—H46B	120.4
H43A—C43A—H43B	109.5	C48B—C47B—C46B	120.6 (4)
C40A—C43A—H43C	109.5	C48B—C47B—H47B	119.7
H43A—C43A—H43C	109.5	C46B—C47B—H47B	119.7
H43B—C43A—H43C	109.5	C49B—C48B—C47B	119.7 (4)
O4A—C44A—C38A	119.1 (4)	C49B—C48B—H48B	120.2
O4A—C44A—C45A	116.1 (4)	C47B—C48B—H48B	120.2
C38A—C44A—C45A	124.0 (4)	C48B—C49B—C50B	120.8 (5)
C46A—C45A—C50A	119.1 (5)	C48B—C49B—H49B	119.6
C46A—C45A—C44A	116.0 (4)	C50B—C49B—H49B	119.6
C50A—C45A—C44A	124.5 (4)	C49B—C50B—C45B	120.1 (4)
C47A—C46A—C45A	120.8 (5)	C49B—C50B—H50B	119.9
C47A—C46A—H46A	119.6	C45B—C50B—H50B	119.9
			
O2A—Ni1—O1A—C1A	−169.3 (4)	C47A—C48A—C49A—C50A	−0.7 (9)
N1A—Ni1—O1A—C1A	13.5 (4)	C48A—C49A—C50A—C45A	0.8 (7)
O1A—Ni1—O2A—C39A	169.5 (4)	C46A—C45A—C50A—C49A	0.3 (7)
N2A—Ni1—O2A—C39A	−7.6 (4)	C44A—C45A—C50A—C49A	172.6 (4)
O1A—Ni1—N1A—C18A	−10.7 (5)	O2B—Ni2—O1B—C1B	−179.4 (3)
N2A—Ni1—N1A—C18A	166.5 (5)	N1B—Ni2—O1B—C1B	1.8 (3)
O1A—Ni1—N1A—C25A	163.8 (4)	O1B—Ni2—O2B—C39B	−156.9 (3)
N2A—Ni1—N1A—C25A	−19.0 (4)	N2B—Ni2—O2B—C39B	21.3 (3)
O2A—Ni1—N2A—C27A	9.8 (4)	O1B—Ni2—N1B—C18B	−1.5 (4)
N1A—Ni1—N2A—C27A	−172.9 (4)	N2B—Ni2—N1B—C18B	−179.6 (3)
O2A—Ni1—N2A—C26A	−177.3 (4)	O1B—Ni2—N1B—C25B	176.4 (3)
N1A—Ni1—N2A—C26A	−0.1 (4)	N2B—Ni2—N1B—C25B	−1.8 (3)
Ni1—O1A—C1A—C2A	172.1 (3)	O2B—Ni2—N2B—C27B	−20.7 (4)
Ni1—O1A—C1A—C6A	−10.4 (7)	N1B—Ni2—N2B—C27B	158.1 (4)
O1A—C1A—C2A—C3A	171.9 (4)	O2B—Ni2—N2B—C26B	160.0 (3)
C6A—C1A—C2A—C3A	−5.7 (7)	N1B—Ni2—N2B—C26B	−21.2 (3)
O1A—C1A—C2A—C11A	−3.8 (7)	Ni2—O1B—C1B—C2B	178.8 (3)
C6A—C1A—C2A—C11A	178.5 (4)	Ni2—O1B—C1B—C6B	−0.6 (6)
C1A—C2A—C3A—C4A	5.1 (8)	O1B—C1B—C2B—C3B	178.7 (4)
C11A—C2A—C3A—C4A	−178.8 (5)	C6B—C1B—C2B—C3B	−1.9 (6)
C2A—C3A—C4A—C5A	−1.3 (7)	O1B—C1B—C2B—C11B	−3.5 (5)
C2A—C3A—C4A—C7C	177.6 (10)	C6B—C1B—C2B—C11B	175.9 (3)
C2A—C3A—C4A—C7A	−173.5 (6)	C1B—C2B—C3B—C4B	0.9 (6)
C3A—C4A—C5A—C6A	−1.7 (7)	C11B—C2B—C3B—C4B	−176.7 (4)
C7C—C4A—C5A—C6A	179.4 (10)	C2B—C3B—C4B—C5B	0.5 (6)
C7A—C4A—C5A—C6A	170.6 (6)	C2B—C3B—C4B—C7B	−179.4 (4)
C4A—C5A—C6A—C1A	0.9 (7)	C3B—C4B—C5B—C6B	−0.9 (6)
C4A—C5A—C6A—C18A	−173.7 (4)	C7B—C4B—C5B—C6B	178.9 (4)
O1A—C1A—C6A—C5A	−174.6 (4)	C4B—C5B—C6B—C1B	0.0 (6)
C2A—C1A—C6A—C5A	2.8 (7)	C4B—C5B—C6B—C18B	−177.8 (4)
O1A—C1A—C6A—C18A	−0.1 (7)	O1B—C1B—C6B—C5B	−179.3 (4)
C2A—C1A—C6A—C18A	177.3 (4)	C2B—C1B—C6B—C5B	1.4 (5)
C3A—C4A—C7A—C8A	−7.1 (11)	O1B—C1B—C6B—C18B	−1.5 (6)
C5A—C4A—C7A—C8A	−179.0 (8)	C2B—C1B—C6B—C18B	179.2 (3)
C3A—C4A—C7A—C9A	114.5 (9)	C5B—C4B—C7B—C10B	1.8 (6)
C5A—C4A—C7A—C9A	−57.4 (11)	C3B—C4B—C7B—C10B	−178.4 (4)
C3A—C4A—C7A—C10A	−125.4 (8)	C5B—C4B—C7B—C9B	121.3 (5)
C5A—C4A—C7A—C10A	62.7 (10)	C3B—C4B—C7B—C9B	−58.8 (5)
C3A—C4A—C7C—C8C	−35 (2)	C5B—C4B—C7B—C8B	−118.7 (5)
C5A—C4A—C7C—C8C	144.3 (17)	C3B—C4B—C7B—C8B	61.1 (5)
C3A—C4A—C7C—C9C	83.8 (19)	C3B—C2B—C11B—O3B	78.6 (5)
C5A—C4A—C7C—C9C	−97.3 (19)	C1B—C2B—C11B—O3B	−99.1 (5)
C3A—C4A—C7C—C10C	−151.5 (16)	C3B—C2B—C11B—C12B	−99.2 (5)
C5A—C4A—C7C—C10C	27 (2)	C1B—C2B—C11B—C12B	83.1 (4)
C3A—C2A—C11A—O3A	−47.0 (7)	O3B—C11B—C12B—C17B	−167.0 (4)
C1A—C2A—C11A—O3A	128.9 (5)	C2B—C11B—C12B—C17B	10.7 (6)
C3A—C2A—C11A—C12A	124.7 (5)	O3B—C11B—C12B—C13B	10.2 (6)
C1A—C2A—C11A—C12A	−59.3 (6)	C2B—C11B—C12B—C13B	−172.1 (3)
O3A—C11A—C12A—C13A	171.4 (5)	C17B—C12B—C13B—C14B	2.3 (6)
C2A—C11A—C12A—C13A	−0.2 (7)	C11B—C12B—C13B—C14B	−175.0 (4)
O3A—C11A—C12A—C17A	−4.3 (7)	C12B—C13B—C14B—C15B	−1.3 (7)
C2A—C11A—C12A—C17A	−175.9 (4)	C13B—C14B—C15B—C16B	−0.1 (7)
C17A—C12A—C13A—C14A	0.9 (7)	C14B—C15B—C16B—C17B	0.4 (7)
C11A—C12A—C13A—C14A	−174.7 (4)	C15B—C16B—C17B—C12B	0.7 (7)
C12A—C13A—C14A—C15A	1.5 (7)	C13B—C12B—C17B—C16B	−2.0 (6)
C13A—C14A—C15A—C16A	−3.1 (7)	C11B—C12B—C17B—C16B	175.1 (4)
C14A—C15A—C16A—C17A	2.4 (7)	C25B—N1B—C18B—C6B	−177.7 (3)
C15A—C16A—C17A—C12A	0.0 (7)	Ni2—N1B—C18B—C6B	0.0 (6)
C13A—C12A—C17A—C16A	−1.6 (7)	C25B—N1B—C18B—C19B	0.2 (5)
C11A—C12A—C17A—C16A	174.1 (4)	Ni2—N1B—C18B—C19B	177.9 (3)
C25A—N1A—C18A—C6A	−169.5 (5)	C5B—C6B—C18B—N1B	179.5 (4)
Ni1—N1A—C18A—C6A	4.5 (7)	C1B—C6B—C18B—N1B	1.8 (6)
C25A—N1A—C18A—C19A	9.9 (7)	C5B—C6B—C18B—C19B	1.6 (5)
Ni1—N1A—C18A—C19A	−176.0 (3)	C1B—C6B—C18B—C19B	−176.1 (3)
C5A—C6A—C18A—N1A	177.5 (5)	N1B—C18B—C19B—C24B	−70.6 (5)
C1A—C6A—C18A—N1A	3.1 (7)	C6B—C18B—C19B—C24B	107.4 (4)
C5A—C6A—C18A—C19A	−1.9 (7)	N1B—C18B—C19B—C20B	108.3 (4)
C1A—C6A—C18A—C19A	−176.4 (4)	C6B—C18B—C19B—C20B	−73.7 (5)
N1A—C18A—C19A—C20A	−104.1 (6)	C24B—C19B—C20B—C21B	0.7 (6)
C6A—C18A—C19A—C20A	75.4 (6)	C18B—C19B—C20B—C21B	−178.2 (4)
N1A—C18A—C19A—C24A	72.9 (6)	C19B—C20B—C21B—C22B	0.0 (7)
C6A—C18A—C19A—C24A	−107.7 (5)	C20B—C21B—C22B—C23B	−0.7 (8)
C24A—C19A—C20A—C21A	0.6 (7)	C21B—C22B—C23B—C24B	0.7 (8)
C18A—C19A—C20A—C21A	177.5 (4)	C20B—C19B—C24B—C23B	−0.7 (7)
C19A—C20A—C21A—C22A	−0.9 (7)	C18B—C19B—C24B—C23B	178.2 (4)
C20A—C21A—C22A—C23A	0.8 (7)	C22B—C23B—C24B—C19B	0.0 (8)
C21A—C22A—C23A—C24A	−0.2 (7)	C18B—N1B—C25B—C26B	−158.1 (3)
C20A—C19A—C24A—C23A	0.0 (7)	Ni2—N1B—C25B—C26B	23.8 (4)
C18A—C19A—C24A—C23A	−177.0 (4)	C27B—N2B—C26B—C25B	−140.3 (4)
C22A—C23A—C24A—C19A	−0.1 (7)	Ni2—N2B—C26B—C25B	39.0 (4)
C18A—N1A—C25A—C26A	−150.8 (5)	N1B—C25B—C26B—N2B	−39.9 (4)
Ni1—N1A—C25A—C26A	34.1 (6)	C26B—N2B—C27B—C34B	−169.9 (4)
N1A—C25A—C26A—N2A	−33.8 (7)	Ni2—N2B—C27B—C34B	10.9 (6)
C27A—N2A—C26A—C25A	−167.2 (5)	C26B—N2B—C27B—C28B	12.4 (6)
Ni1—N2A—C26A—C25A	19.1 (7)	Ni2—N2B—C27B—C28B	−166.7 (3)
C26A—N2A—C27A—C34A	−177.9 (5)	N2B—C27B—C28B—C29B	63.6 (5)
Ni1—N2A—C27A—C34A	−5.5 (7)	C34B—C27B—C28B—C29B	−114.1 (4)
C26A—N2A—C27A—C28A	1.9 (7)	N2B—C27B—C28B—C33B	−117.0 (4)
Ni1—N2A—C27A—C28A	174.4 (3)	C34B—C27B—C28B—C33B	65.2 (5)
N2A—C27A—C28A—C33A	93.9 (5)	C33B—C28B—C29B—C30B	−1.0 (6)
C34A—C27A—C28A—C33A	−86.3 (5)	C27B—C28B—C29B—C30B	178.4 (4)
N2A—C27A—C28A—C29A	−81.2 (6)	C28B—C29B—C30B—C31B	0.0 (7)
C34A—C27A—C28A—C29A	98.7 (5)	C29B—C30B—C31B—C32B	1.4 (7)
C33A—C28A—C29A—C30A	0.2 (7)	C30B—C31B—C32B—C33B	−2.0 (7)
C27A—C28A—C29A—C30A	175.3 (4)	C31B—C32B—C33B—C28B	1.1 (7)
C28A—C29A—C30A—C31A	−1.5 (8)	C29B—C28B—C33B—C32B	0.4 (6)
C29A—C30A—C31A—C32A	2.1 (8)	C27B—C28B—C33B—C32B	−178.9 (4)
C30A—C31A—C32A—C33A	−1.4 (8)	N2B—C27B—C34B—C35B	−177.7 (4)
C29A—C28A—C33A—C32A	0.5 (7)	C28B—C27B—C34B—C35B	0.0 (6)
C27A—C28A—C33A—C32A	−174.6 (4)	N2B—C27B—C34B—C39B	5.4 (6)
C31A—C32A—C33A—C28A	0.2 (7)	C28B—C27B—C34B—C39B	−176.9 (3)
N2A—C27A—C34A—C35A	173.5 (4)	C39B—C34B—C35B—C36B	1.8 (6)
C28A—C27A—C34A—C35A	−6.4 (6)	C27B—C34B—C35B—C36B	−175.3 (4)
N2A—C27A—C34A—C39A	−4.3 (7)	C34B—C35B—C36B—C37B	−3.6 (6)
C28A—C27A—C34A—C39A	175.8 (4)	C34B—C35B—C36B—C40B	174.7 (4)
C39A—C34A—C35A—C36A	−1.8 (6)	C35B—C36B—C37B—C38B	3.7 (6)
C27A—C34A—C35A—C36A	−179.7 (4)	C40B—C36B—C37B—C38B	−174.5 (4)
C34A—C35A—C36A—C37A	−2.5 (6)	C36B—C37B—C38B—C39B	−2.1 (6)
C34A—C35A—C36A—C40A	176.4 (4)	C36B—C37B—C38B—C44B	171.0 (4)
C35A—C36A—C37A—C38A	1.8 (6)	Ni2—O2B—C39B—C34B	−12.6 (5)
C40A—C36A—C37A—C38A	−177.2 (4)	Ni2—O2B—C39B—C38B	165.7 (3)
C36A—C37A—C38A—C39A	3.4 (6)	C35B—C34B—C39B—O2B	178.4 (4)
C36A—C37A—C38A—C44A	−175.3 (4)	C27B—C34B—C39B—O2B	−4.6 (6)
Ni1—O2A—C39A—C34A	1.2 (6)	C35B—C34B—C39B—C38B	0.1 (6)
Ni1—O2A—C39A—C38A	−177.0 (3)	C27B—C34B—C39B—C38B	177.1 (3)
C35A—C34A—C39A—O2A	−171.3 (4)	C37B—C38B—C39B—O2B	−178.3 (4)
C27A—C34A—C39A—O2A	6.5 (7)	C44B—C38B—C39B—O2B	8.6 (6)
C35A—C34A—C39A—C38A	6.9 (6)	C37B—C38B—C39B—C34B	0.1 (6)
C27A—C34A—C39A—C38A	−175.3 (4)	C44B—C38B—C39B—C34B	−173.0 (3)
C37A—C38A—C39A—O2A	170.7 (4)	C35B—C36B—C40B—C43B	−179.3 (4)
C44A—C38A—C39A—O2A	−10.9 (7)	C37B—C36B—C40B—C43B	−1.1 (6)
C37A—C38A—C39A—C34A	−7.6 (6)	C35B—C36B—C40B—C42B	58.9 (5)
C44A—C38A—C39A—C34A	170.9 (4)	C37B—C36B—C40B—C42B	−123.0 (4)
C37A—C36A—C40A—C43A	16.8 (6)	C35B—C36B—C40B—C41B	−60.2 (5)
C35A—C36A—C40A—C43A	−162.1 (4)	C37B—C36B—C40B—C41B	118.0 (4)
C37A—C36A—C40A—C41A	136.6 (4)	C37B—C38B—C44B—O4B	−135.2 (4)
C35A—C36A—C40A—C41A	−42.3 (5)	C39B—C38B—C44B—O4B	38.1 (6)
C37A—C36A—C40A—C42A	−103.9 (4)	C37B—C38B—C44B—C45B	41.2 (5)
C35A—C36A—C40A—C42A	77.2 (5)	C39B—C38B—C44B—C45B	−145.6 (4)
C37A—C38A—C44A—O4A	−28.5 (6)	O4B—C44B—C45B—C46B	−145.3 (4)
C39A—C38A—C44A—O4A	153.0 (4)	C38B—C44B—C45B—C46B	38.2 (6)
C37A—C38A—C44A—C45A	140.5 (4)	O4B—C44B—C45B—C50B	29.4 (6)
C39A—C38A—C44A—C45A	−38.0 (6)	C38B—C44B—C45B—C50B	−147.1 (4)
O4A—C44A—C45A—C46A	56.9 (6)	C50B—C45B—C46B—C47B	−0.3 (7)
C38A—C44A—C45A—C46A	−112.4 (5)	C44B—C45B—C46B—C47B	174.3 (4)
O4A—C44A—C45A—C50A	−115.7 (5)	C45B—C46B—C47B—C48B	1.0 (7)
C38A—C44A—C45A—C50A	75.0 (6)	C46B—C47B—C48B—C49B	0.0 (8)
C50A—C45A—C46A—C47A	−1.4 (8)	C47B—C48B—C49B—C50B	−1.7 (8)
C44A—C45A—C46A—C47A	−174.4 (5)	C48B—C49B—C50B—C45B	2.4 (8)
C45A—C46A—C47A—C48A	1.5 (10)	C46B—C45B—C50B—C49B	−1.4 (7)
C46A—C47A—C48A—C49A	−0.4 (10)	C44B—C45B—C50B—C49B	−176.2 (4)

### Hydrogen-bond geometry (Å, º)

*Cg*2, *Cg*4, *Cg*8, *Cg*11, *Cg*13, *Cg*14 and *Cg*17 are the centroids of the Ni1/O1*A*/C1*A*/C6*A*/C18*A*/N1*A*, C1*A*–C6*A*, C34*A*–C39*A*, Ni2/O1*B*/C1*B*/C6*B*/C18*B*/N1*B*, C1*B*–C6*B*, C12*B*–C17*B* and C34*B*–C39*B* rings, respectively.

**Table:**

*D*—H···*A*	*D*—H	H···*A*	*D*···*A*	*D*—H···*A*
C50*A*—H50*A*···O1*A*	0.95	2.55	3.354 (5)	143
C25*A*—H25*B*···O2*A*^i^	0.99	2.40	3.394 (7)	178
C24*B*—H24*B*···O4*A*	0.95	2.58	3.343 (6)	138
C25*B*—H25*C*···O4*A*	0.99	2.35	3.336 (5)	171
C26*B*—H26*C*···O1*B*^ii^	0.99	2.45	3.294 (5)	143
C26*B*—H26*C*···O2*B*^ii^	0.99	2.45	3.309 (5)	144
C25*B*—H25*D*···O4*B*^ii^	0.99	2.45	3.283 (4)	141
C10*A*—H10*C*···*Cg*2^iii^	0.98	2.98	3.771 (12)	138
C15*B*—H15*B*···*Cg*11^iv^	0.95	2.74	3.593 (5)	150
C20*A*—H20*A*···*Cg*8^i^	0.95	2.77	3.424 (5)	127
C20*B*—H20*B*···*Cg*17^ii^	0.95	2.71	3.585 (5)	153
C33*A*—H33*A*···*Cg*4^i^	0.95	2.79	3.623 (5)	146
C33*B*—H33*B*···*Cg*13^ii^	0.95	2.66	3.465 (4)	143
C43*A*—H43*B*···*Cg*14^iv^	0.98	2.60	3.457 (5)	147

Symmetry codes: (i) −*x*+2, −*y*+1, −*z*+1; (ii) −*x*+1, −*y*, −*z*; (iii) −*x*+1, −*y*+1, −*z*+1; (iv) −*x*+2, −*y*, −*z*.
